# The Role of Indoleamine 2,3-Dioxygenase in Diethylnitrosamine-Induced Liver Carcinogenesis

**DOI:** 10.1371/journal.pone.0146279

**Published:** 2016-01-04

**Authors:** Yuhei Shibata, Takeshi Hara, Junji Nagano, Nobuhiko Nakamura, Tomohiko Ohno, Soranobu Ninomiya, Hiroyasu Ito, Takuji Tanaka, Kuniaki Saito, Mitsuru Seishima, Masahito Shimizu, Hisataka Moriwaki, Hisashi Tsurumi

**Affiliations:** 1 First Departments of Internal Medicine, Gifu University Graduate School of Medicine, Gifu, Japan; 2 Departments of Informative Clinical Medicine, Gifu University Graduate School of Medicine, Gifu, Japan; 3 Departments of Tumor Pathology, Gifu University Graduate School of Medicine, Gifu, Japan; 4 Human Health Sciences, Graduate School of Medicine and Faculty of Medicine, Kyoto University, Kyoto, Japan; University of Navarra School of Medicine and Center for Applied Medical Research (CIMA), SPAIN

## Abstract

Indoleamine 2,3-dioxygenase (IDO), a tryptophan-catabolizing intracellular enzyme of the L-kynurenine pathway, causes preneoplastic cells and tumor cells to escape the immune system by inducing immune tolerance; this mechanism might be associated with the development and progression of human malignancies. In the present study, we investigated the role of IDO in diethylnitrosamine (DEN)-induced hepatocarcinogenesis by using IDO-knockout (KO) mice. To induce hepatocellular carcinoma (HCC), hepatic adenoma, and preneoplastic hepatocellular lesions termed foci of cellular alteration (FCA), male IDO-wild-type (WT) and IDO-KO mice with a C57BL/6J background received a single intraperitoneal injection of DEN at 2 weeks of age. The mice were sacrificed to evaluate the development of FCA and hepatocellular neoplasms. HCC overexpressed IDO and L-kynurenine compared to surrounding normal tissue in the DEN-treated IDO-WT mice. The number and cell proliferative activity of FCAs, and the incidence and multiplicity of HCC were significantly greater in the IDO-WT than in the IDO-KO mice. The expression levels of the IDO protein, of L-kynurenine, and of IFN-γ, COX-2, TNF-α, and Foxp3 mRNA were also significantly increased in the DEN-induced hepatic tumors that developed in the IDO-WT mice. The mRNA expression levels of CD8, perforin and granzyme B were markedly increased in hepatic tumors developed in IDO-KO mice. Moreover, Foxp3-positive inflammatory cells had infiltrated into the livers of DEN-treated IDO-WT mice, whereas fewer cells had infiltrated into the livers of IDO-KO mice. Induction of IDO and elevation of L-kynurenine might play a critical role in both the early and late phase of liver carcinogenesis. Our findings suggest that inhibition of IDO might offer a promising strategy for the prevention of liver cancer.

## Introduction

Hepatocellular carcinoma (HCC), the dominant form of primary liver carcinoma, is a serious healthcare problem worldwide because of its increasing morbidity and high mortality [1]. Chronic inflammation and subsequent cirrhosis of the liver, which is mostly induced by persistent infection with hepatitis viruses, contribute to the development of HCC. Therefore, the immune system, which regulates infection and inflammation in the liver, is profoundly associated with liver carcinogenesis [2]. In addition, tumor immune escape, which is highly associated with tumor antigen-specific immune tolerance as well as with the tumor immunosuppressive microenvironment, might play a role in the initiation and progression of HCC [3, 4]. However, the precise mechanisms by which abnormalities in tumor immunity, such as unresponsiveness of the immune system, contribute to liver carcinogenesis are not fully understood.

Tumor cells express many antigens to which the immune system can respond. Hosts possess T cells that are specific to these antigens and that act to reject the tumor. However, during tumor progression, the host fails to reject tumors that have evolved ways to escape immune surveillance [5]. Recent studies have suggested that indoleamine 2,3-dioxygenase (IDO) plays a crucial role in the induction of immune tolerance to tumors [6, 7]. IDO is an intracellular enzyme that catalyzes the first and rate-limiting step in the catabolism of the essential amino acid tryptophan along the kynurenine pathway [8]. The regulation of IDO expression is mediated by several immune and inflammatory factors, including interferon (IFN)-γ and cyclooxygenase (COX)-2 [9]. In the tumor microenvironment, increased IDO activity inhibits cell proliferation and induces apoptosis in T cells and natural killer cells through tryptophan depletion and production of toxic tryptophan catabolites [10, 11]. In addition, IDO increases immunosuppressive regulatory T-cell (Tregs) activity, which also contributes to the promotion of immune tolerance in cancer tissue [12].

IDO overexpression is correlated with poor clinical outcome in patients with ovarian, endometrial, and colorectal carcinomas [13, 14]. We have previously reported that IDO expression in tumor cells and the serum concentration of kynurenine in patients with diffuse large B-cell lymphoma [15–17], or with acute myeloid leukemia [18, 19] are useful prognostic factors. On the other hand, several preclinical studies have demonstrated that IDO inhibitors, such as 1-methyl-tryptophan (1-MT), are therapeutically beneficial for inhibition of cancer cell growth, especially when they are combined with different types of cytotoxic chemotherapeutic agents [20, 21]. We have also reported that IDO up-regulation accelerates chemically induced colorectal carcinogenesis in rats, whereas supplementation with 1-MT suppresses this carcinogenesis by inhibiting the expression and activation of IDO [22, 23]. These reports suggest that targeting IDO might be an effective strategy for the treatment and the prevention of certain types of human malignancies.

Recently, Pan K *et al*. [24] reported that IDO was overexpressed in 35.5% of HCC resection samples and resulted in significantly poor prognosis. Systemic IDO activity is enhanced in chronic hepatitis C patients in correlation with the degree of liver inflammation and fibrosis [25]. We have also shown that IDO plays a critical role in the regulation of liver immunity and inflammation [26, 27]. These reports implicate abnormalities in the expression and activity of IDO in the initiation and promotion of HCC, whereas the precise role of this enzyme in hepatocarcinogenesis has not yet been clarified. In the present study, we investigated the role of IDO in diethylnitrosamine (DEN)-induced liver carcinogenesis by using IDO-deficient mice to obtain preclinical data for therapy and/or chemoprevention of HCC by modulating IDO expression.

## Materials and Methods

### Animals and chemicals

*IDO1* gene-knockout (KO) mice on a C57BL/6J background were obtained from the Jackson Laboratory (Bar Harbor, ME, USA). C57BL/6J mice from Japan SLC (Shizuoka, Japan) were used as control IDO-wild-type (WT) mice. IDO-WT and IDO-KO mice were selected from the offspring of heterozygous/homozygous mating by performing PCR on tail DNA. DEN was purchased from Sigma-Aldrich (St Louis, MO, USA). All mice were maintained at the Gifu University Animal Facility according to Institutional Animal Care Guidelines and housed in plastic cages with free access to drinking water and a pelleted basal diet (CRF-1; Oriental Yeast, Tokyo, Japan).

### Experimental procedures

The animal experiments were approved by the Committee of Institutional Animal Experiments of Gifu University [28]. A total of 39 male IDO-KO mice and 35 male IDO-WT mice were used in the present study. At 2 weeks of age, 28 IDO-KO mice and 25 IDO-WT mice were given a single intraperitoneal injection of DEN (25 mg/kg body weight) to induce hepatic neoplasms (HCC and liver cell adenoma) and preneoplastic lesions known as foci of cellular alteration (FCA). The remaining 11 IDO-KO mice and 10 IDO-WT mice received an injection of saline and constituted the DEN-untreated control groups. At 20 and 32 weeks of age, animals were sacrificed by CO_2_ asphyxiation to determine the incidence and multiplicity of FCA and liver cell neoplasms. After careful macroscopic inspection at sacrifice, the livers were removed and fixed overnight in 10% buffered formalin. Paraffin-embedded sections of livers were prepared by using routine procedures for histopathological and immunohistochemical examinations.

### Histopathological and immunohistochemical analyses

Maximum sagittal sections of each liver lobe (six sublobes) from each mouse were used for histopathological examination. Four μm-thick sections of formalin-fixed and paraffin-embedded livers were prepared and stained with hematoxylin and eosin (H&E) for histopathology. The presence of FCA was determined according to the criteria described by Frith *et al*. [29]. The multiplicity of FCA were assessed on a per unit area (/cm^2^) basis.

Immunohistochemical analyses for IDO and L-kynurenine were performed with anti-IDO (LYFESPAN, Seattle, WA, USA) and anti-L-kynurenine (Abnova, Taipei City, Taiwan) antibodies, respectively [22]. Tissue sections (5 μm thick) were cut, deparaffinized, placed in 10 mmol/l citrate buffer (pH 6.0), and boiled at 121°C for 15 min. Endogenous peroxidase activity was blocked by incubating the sections in 0.3% H2O2 for 15 min. After three washes with 50 mmol/l phosphate buffer (pH 7.6), the sections were preincubated with 2% bovine serum albumin in PBS for 10 min at room temperature and were then incubated with the primary antibodies, anti-β-catenin (1:1000; BD Biosciences PharMingen, San Diego, CA, USA), anti-IDO (1:1000; LYFESPAN, Seattle, WA, USA) and anti-L-Kynurenine (1:1000; Abnova, Taipei City, Taiwan), overnight at 4°C. Subsequently, the sections for β-catenin and IDO analysis were incubated with biotinylated secondary antibodies against the primary antibodies (1:400; DAKO Corp., Carpinteria, CA, USA) for 30 min followed by incubation with avidin-coupled peroxidase (DAKO) for 10 min at room temperature. The sections for L-Kynurenine analysis were incubated with peroxidase-labeled polymer-conjugated secondary antibodies against the primary antibodies for 30 min at room temperature. The sections were then developed with 3,3’-diaminobenzidine (DAB) using the DAKO Liquid DAB Substrate-Chromogen System (DAKO) and were counterstained with hematoxylin. The sections were finally dehydrated and coverslipped. Forkhead box protein 3 (Foxp3) was immunohistochemically analyzed by using an anti-Foxp3 antibody (eBioscience, San Diego, CA, USA) [30]. Immunohistochemical staining of proliferating cell nuclear antigen (PCNA), a G_1_-to-S phase marker, was performed to estimate the cell proliferative activity of FCA by using an anti-PCNA antibody (Santa Cruz Biotechnology, Santa Cruz, CA, USA) [28]. On the PCNA-immunostained sections, the cells with intensively reacted nuclei were considered to be positive for PCNA, and the indices (%) were calculated in 20 FCA randomly selected from each IDO-WT and IDO-KO mouse that was sacrificed at 32 weeks of age [31].

### Quantitative real-time reverse transcription-polymerase chain reaction

Total RNA was extracted from tumor tissue and from non-cancerous tissues of the liver by using the RNeasy Mini Kit (Qiagen, Hilden, Germany) [31]. Total RNA (1 μg) was used for the synthesis of the first strand cDNA. Quantitative real-time reverse transcription-polymerase chain reaction (RT-PCR) was performed by using the TOYOBO real-time PCR Master Mix (TOYOBO, Osaka, Japan) and specific primer/probe sets that amplify IDO, tryptophan 2,3-dioxygenase (TDO), IFN-γ, COX-2, tumor necrosis factor (TNF)- α, Foxp3, CD8, FasL, perforin, granzyme B and GAPDH genes (TaqMan Gene Expression Assays; Applied Biosystems, Foster City, CA). Each sample was analyzed using a LightCycler 1.0 instrument (Roche Diagnostics, Indianapolis, IN, USA) [32]. The expression level of each gene was normalized to the GAPDH expression level by using the standard curve method.

### Determination of serum tryptophan and kynurenine concentrations

After serum samples were mixed with three volumes of 3% perchloric acid and centrifuged, tryptophan and kynurenine concentrations in the supernatant were measured using high-performance liquid chromatography (HPLC), as described previously [33].

### Statistical analysis

All data points expect for the incidence of tumors are expressed as the mean ± SD. Statistical significance was evaluated by using the Tukey-Kramer multiple comparison test or Fisher’s exact probability test. *P* values less than 0.05 were considered to indicate statistical significance.

## Results

### General observations of the IDO- and KO mice

At 20 weeks of age, the values for body or liver weights of the IDO- or KO-mice injected with saline or DEN did not significantly differ among the groups. At 32 weeks of age, the mean body (*P* < 0.01) and liver (*P* < 0.05) weight of the IDO-KO mice were significantly lower than those of the IDO-WT mice when the mice were treated with DEN, but the mean relative liver weights of the four groups were comparable (Table 1).

**Table 1:** Body and liver weights of the experimental mice.

		20 weeks of age				32 weeks of age			
	Mice	No. of	Body	Liver	Relative Liver weight	No. of	Body	Liver	Relative Liver weight
Treatment	type	mice	Weight (g)	weight	(g/100 g body weight)	mice	weight (g)	weight	(g/100g body weight)
saline	Wild	4	29.8±1.6^a^	1.2±0.2	4.0±0.5	6	36.3±2.0	1.6±0.2	4.5±0.3
saline	IDO(-/-)	4	31.3±0.9	1.2±0.2	3.8±0.7	7	32.9±5.3	1.4±0.4	4.3±0.6
DEN	Wild	12	32.0±2.0	1.4±0.2	4.4±0.6	13	34.5±1.5 b	1.7±0.2 c	4.8±0.6
DEN	IDO(-/-)	14	30.9±2.3	1.4±0.2	4.4±0.5	14	30.4±3.1	1.4±0.3	4.4±0.6

a Mean±SD.

b Significantly different from DEN-IDO(-/-) group by Tukey-Kramer multiple comparison test (p<0.01)

c Significantly different from DEN-IDO(-/-) group by Tukey-Kramer multiple comparison test (p<0.05)

### Development of FCA and proliferative activity in FCA

**Fig 1:**
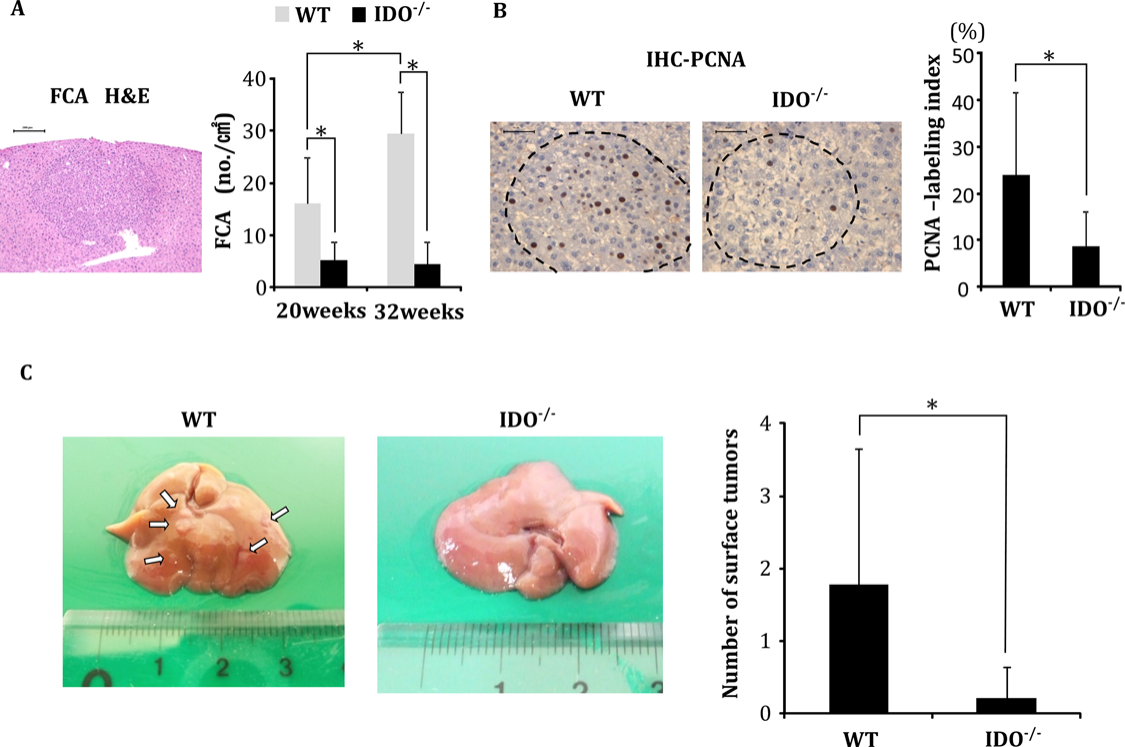
Analysis of pre-neoplastic lesions and nodules of DEN-treated mice. Development of foci of cellular alteration (FCA), the proliferating cell nuclear antigen (PCNA)-labeling indices of the FCA, and the nodule frequency on the liver surface of DEN-treated experimental mice. (A) A representative photograph of a FCA that developed in DEN-treated WT mice at 32 weeks of age (H&E staining, left panel) and the average number of FCA in DEN-treated WT and IDO-/- mice at 20 and 32 weeks of age (right panel). (B) Representative photographs of PCNA-immunohistochemical analysis of the FCA that developed in DEN-treated WT mice and IDO-/- mice at 32 weeks of age (left panels). The PCNA-labeling indices of the FCA that developed in the experimental mice were determined by counting the number of PCNA-positive nuclei in the FCA (right panel). (C) Tumor nodules on the liver surface of DEN-treated WT and IDO-/- mice at 32 weeks. The white arrows indicate tumor nodules (left panel). The number of liver surface tumors in IDO-/- mice was significantly lower than that in wild-type mice (right panel). *, P<0.01 Data points represent the mean ± SD.

At the time of euthanasia (20 and 32 weeks of age), FCA had developed in both IDO-WT and IDO-KO mice that had received DEN. However, the mean number of FCA in the IDO-WT mice was significantly greater than that in the IDO-KO mice both at 20 and 32 weeks of age (Fig 1A, *P* < 0.01). Furthermore, in IDO-WT mice that had received DEN, the mean number of FCA at 32 weeks of age was significantly higher than that at 20 weeks of age (*P* < 0.01), suggesting that FCA development was markedly accelerated by the presence of the *IDO1* gene. There were no microscopically observable changes at the termination of the experiment (32 weeks of age), including FCA and hepatocellular neoplasms, in either the IDO-WT or the IDO-KO mice that received saline alone.

Cell proliferation activity of DEN-induced FCA in the IDO-WT and IDO-KO mice was determined at 32 weeks using PCNA-immunohistochemistry. The mean PCNA-labeling index in the FCA of IDO-WT mice was significantly higher than that in the IDO-KO mice (Fig 1B, *P* < 0.01), indicating that progression of liver tumorigenesis in the IDO-WT mice might be associated with accelerated cell proliferation.

### Development of hepatocellular neoplasms

When the mice were treated with DEN and sacrificed at 32 weeks of age, multiple tumors, which were histopathologically diagnosed as liver cell adenoma or HCC, had developed on the hepatic surface of the IDO-WT mice, while only a few tumors had developed in IDO-KO mice (Fig 1C). The mean number of tumors on the hepatic surface of the IDO-WT mice was significantly higher than that of the IDO-KO mice (Fig 1C, *P* < 0.01). Macroscopic liver tumors did not develop in the IDO-WT or IDO-KO mice that received saline (data not shown).

Microscopic analyses indicated that liver cell adenomas had developed in both DEN-treated IDO-WT and IDO-KO mice at 32 weeks (Table 2). The incidence and multiplicity of liver cell adenomas in IDO-WT mice were higher than those in IDO-KO mice, but the differences did not reach statistical significance. The incidence and multiplicity of HCC development in DEN-treated IDO-WT mice were 31% and 0.85 ± 1.46, respectively, whereas no HCC developed in the DEN-treated IDO-KO mice (*P* < 0.05). Among the DEN-treated groups, the multiplicity of total liver neoplasms in the IDO-WT mice was also significantly higher than that in the IDO-KO mice (*P* < 0.05).

**Table 2:** Incidence and multiplicity of hepatic neoplasms and FCA in the experimental mice (32 weeks-groups).

Treatment			Incidence		Multiplicity (no. of neoplasms/mouse)		
	Mice type	No. mice examined	Adenoma	HCC	Total	Adenoma	HCC
saline	Wild	6	0/6 (0%)	0/6 (0%)	0	0	0
saline	IDO(-/-)	7	0/7 (0%)	0/7 (0%)	0	0	0
DEN	Wild	13	6/13 (46%)	4/13 (31%) b	1.85±2.94 a,c	1.00±1.68	0.85±1.46 c
DEN	IDO(-/-)	14	2/14 (14%)	0/14 (0%)	0.14±0.36	0.14±0.36	0

a Mean±SD.

b p<0.05 (Significantly different from DEN (-/-) group by Fisher's exact probability test)

c p<0.05 (Significantly different from DEN (-/-) group by Tukey-Kramer multiple comparison test)

### Immunohistochemistry of IDO and L-kynurenine in HCC and liver cell adenomas

**Fig 2:**
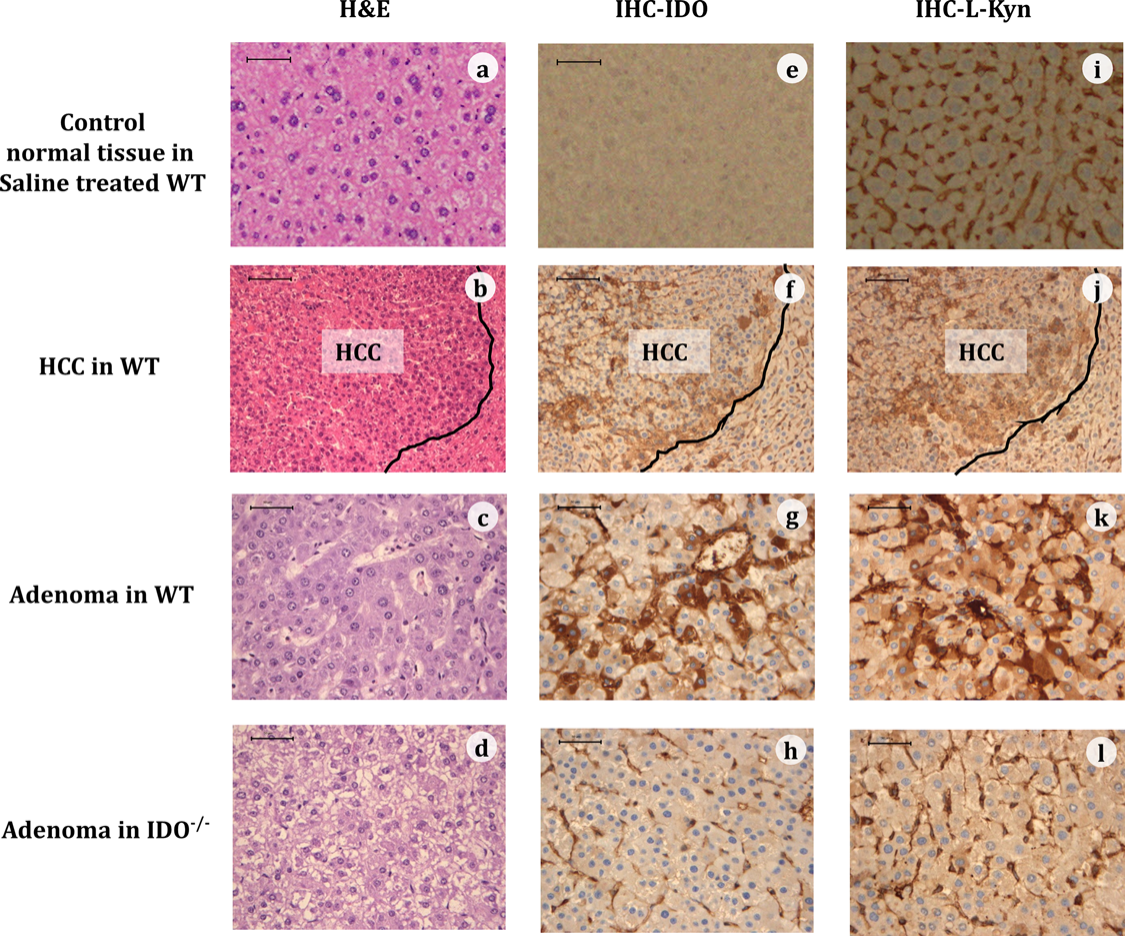
Representative immunohistochemical expression (IHC) of IDO and kynurenine (KYN). Representative images of normal tissue (top panel) of a saline treated WT mouse, of HCC (second panels) and adenoma (third panels) of a WT mouse, and of adenoma of an IDO-/- mouse (bottom panels), at 32 weeks of DEN treatment that were stained with H&E (a-d), or were immunohistochemically stained for the IDO protein (e-h) or for L-KYN (i-l). The black line in the HCC tissues demarcates HCC from non-HCC tissue. (Bar = 50 μm)

The expression levels of IDO and L-kynurenine in HCCs and hepatic adenomas were examined by immunohistochemical analyses at 32 weeks (Fig 2). In the livers of DEN-treated IDO-WT mice, the expression levels of IDO and L-kynurenine were obviously higher in HCCs as compared to the surrounding non-cancerous tissue. The expression levels of IDO and L-kynurenine in adenomas were also increased compared to the surrounding non-cancerous tissue in IDO-WT mice, whereas the expression levels of these molecules were markedly lower in IDO-KO mice compared with IDO-WT mice. These findings, together with those of Table 2, suggested that induction of IDO and elevation of L-kynurenine might be associated with DEN-induced liver tumorigenesis.

### Expression of IFN-γ, COX-2, and TNF-α mRNA in non-tumorous liver tissues and hepatic tumors

**Fig 3:**
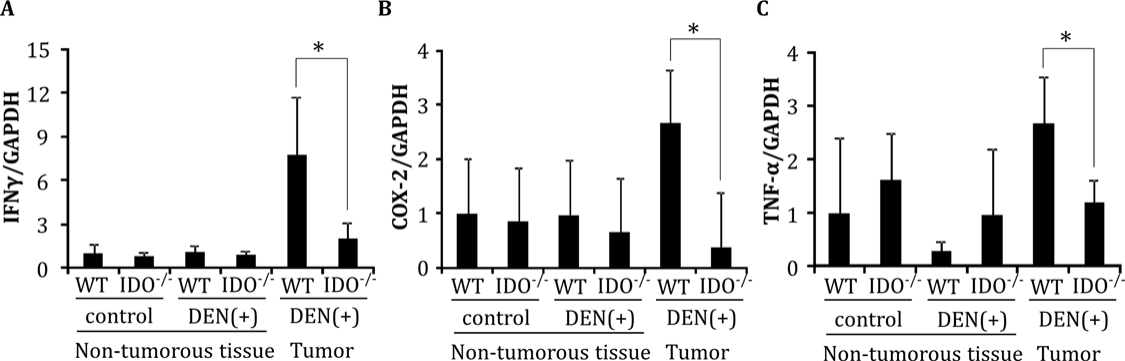
Gene expression of IFN-γ, COX-2, and TNF-α in the liver. Expression levels of (A) IFN-γ, (B) COX-2, and (C) TNF-α mRNA in hepatic tumors and in normal liver tissues of the indicated mice were measured using quantitative RT-PCR. Data points represent the mean ± SD. *, *p*<0.05.

Quantitative RT-PCR analyses showed that the mRNA expression levels of IFN-γ, COX-2, and TNF-α, which are regulated by inflammatory cells and are implicated in the induction of IDO [34, 35], in DEN-induced hepatic tumors in IDO-WT mice were significantly higher than their expression levels in IDO-KO mice at 32 weeks (Fig 3, *P* < 0.05). In non-tumorous liver tissues of IDO-WT and IDO-KO mice, there were no significant differences in the expression levels of these mRNAs, irrespective of DEN treatment (Fig 3).

### Expression levels of Foxp3 in non-tumorous liver tissues, and hepatic tumors

**Fig 4:**
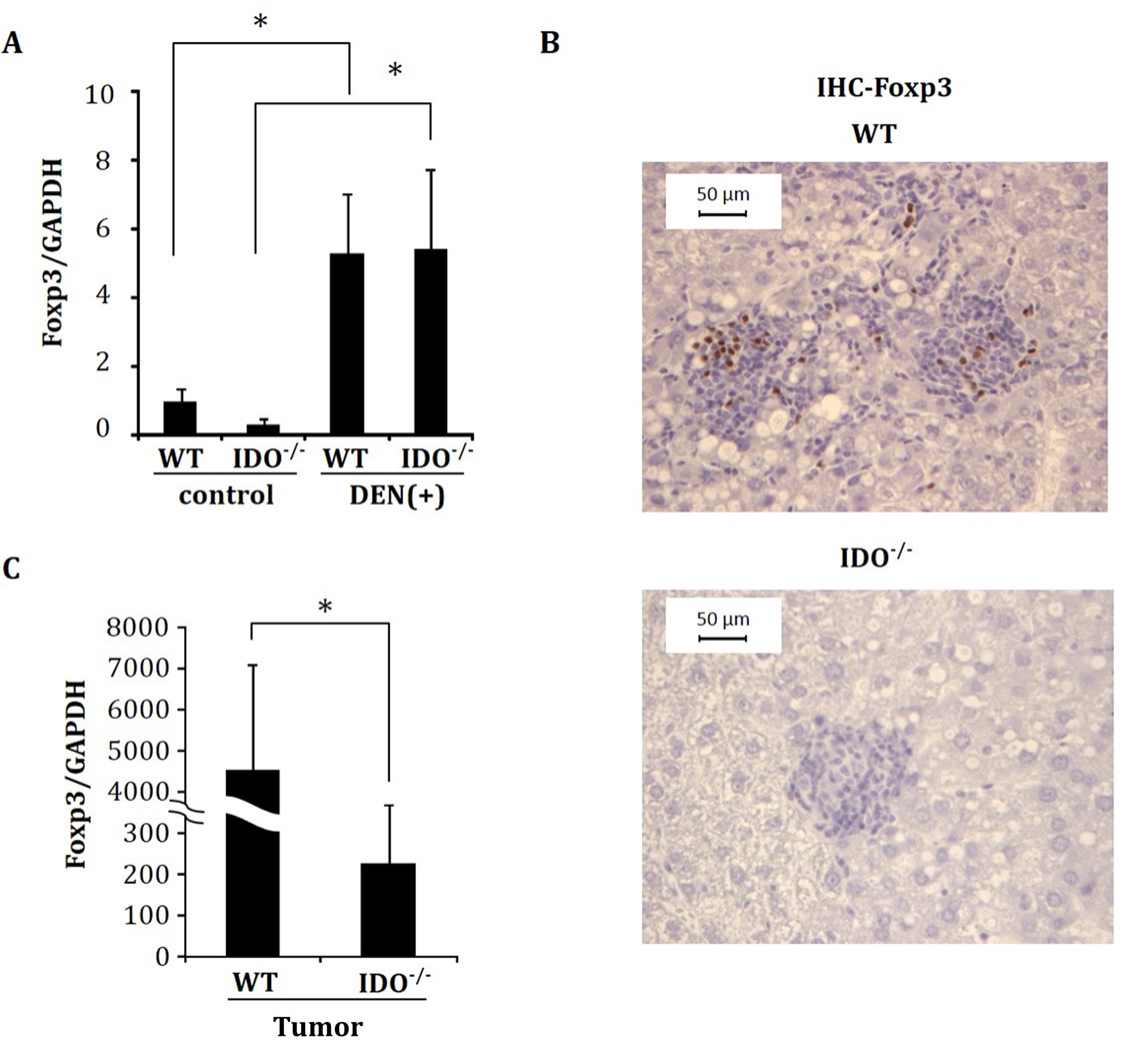
Expression of Foxp3 analyzed by immunohistochemistry and RT-PCR. (A) Gene expression of Foxp3 in control and DEN-treated non-tumorous liver tissues at 32 weeks. (B) Representative pictures of the immunohistochemical analysis of FCA for Foxp3 and (C) Foxp3 mRNA expression in hepatic tumors of DEN-treated mice. mRNA expression was determined using quantitative RT-PCR. Data points represent the mean ± SD. *, p<0.05.

Induction of Tregs by IDO promotes the escape of pre-malignant and malignant cells from antitumor immune responses [36]. Therefore, the expression levels of Foxp3, which is one of the most distinctive markers of Tregs [37], were next examined in the non-tumorous liver tissues and hepatic tumors of experimental mice. Irrespective of genotype, the hepatic expression levels of Foxp3 mRNA were significantly increased by injection with DEN (Fig 4A). Immunohistochemical analyses also showed that, in comparison to IDO-KO mice, there was a marked infiltration of Foxp3-positive inflammatory cells in the livers of IDO-WT mice when the mice received DEN (Fig 4B). Moreover, the expression levels of Foxp3 mRNA were markedly increased, by approximately 20-fold, in hepatic tumors in IDO-WT mice as compared to IDO-KO mice (Fig 4C, *P* < 0.05).

### Expression levels of CD8, FasL, perforin and granzyme B in non-tumorous liver tissues and hepatic tumors

**Fig 5:**
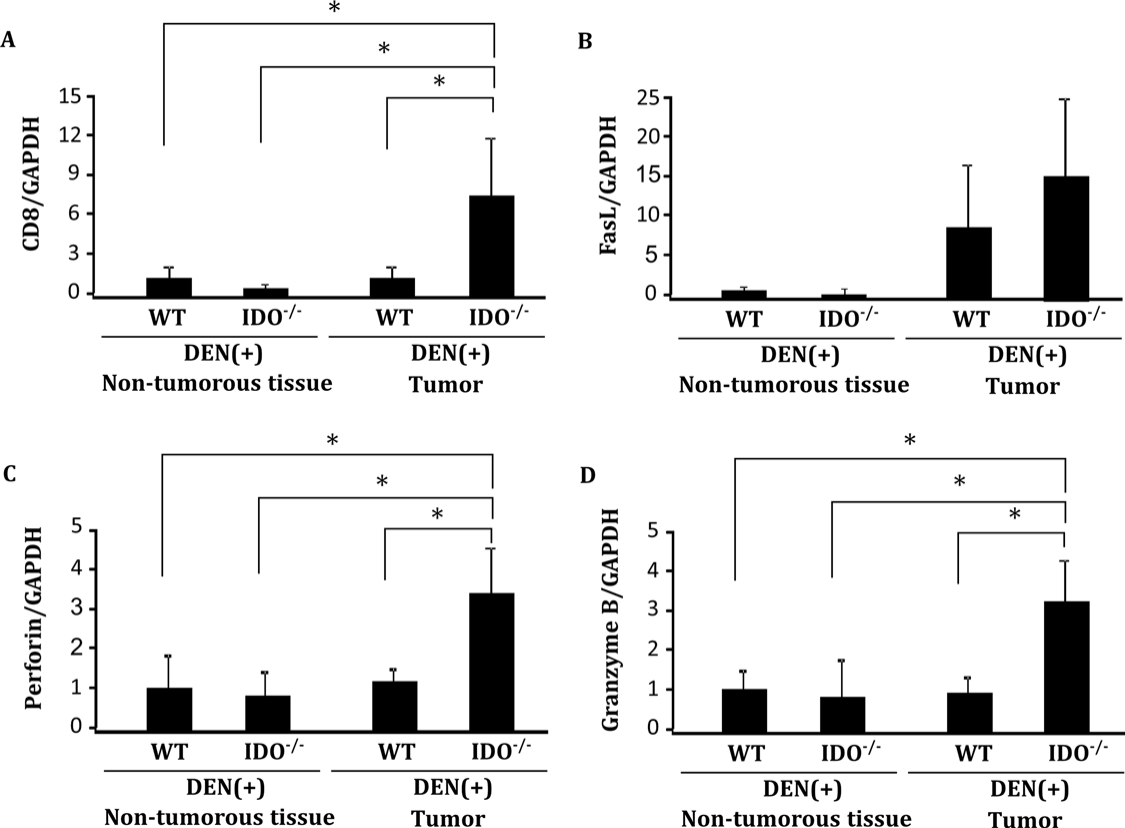
Expression levels of CD8, FasL, perforin and granzyme B in non-tumorous liver tissues and hepatic tumors. Expression levels of (A) CD8, (B) FasL, (C) perforin and (D) granzyme B mRNA in hepatic tumors and in normal liver tissues of the indicated mice were measured using quantitative RT-PCR. Data points represent the mean ± SD. *, *p*<0.05.

In order to evaluate the activity of tumor specific cytotoxic T lymphocytes, we analyzed the hepatic expression levels of CD8, FasL, perforin and granzyme B, which are cytotoxic molecules used by CD8+ T lymphocytes, by performed quantitative RT-PCR analysis. The mRNA expression levels of CD8, perforin, and granzyme B were markedly increased in hepatic tumors in IDO-KO mice as compared to IDO-WT mice (Fig 5A, 5C and 5D, *P* < 0.05). There was no significant difference in FasL mRNA expression in hepatic tumors between IDO-KO mice and IDO-WT mice.

### Serum L-kynurenine/L-tryptophan ratio and hepatic expression of IDO and TDO mRNA

**Fig 6:**
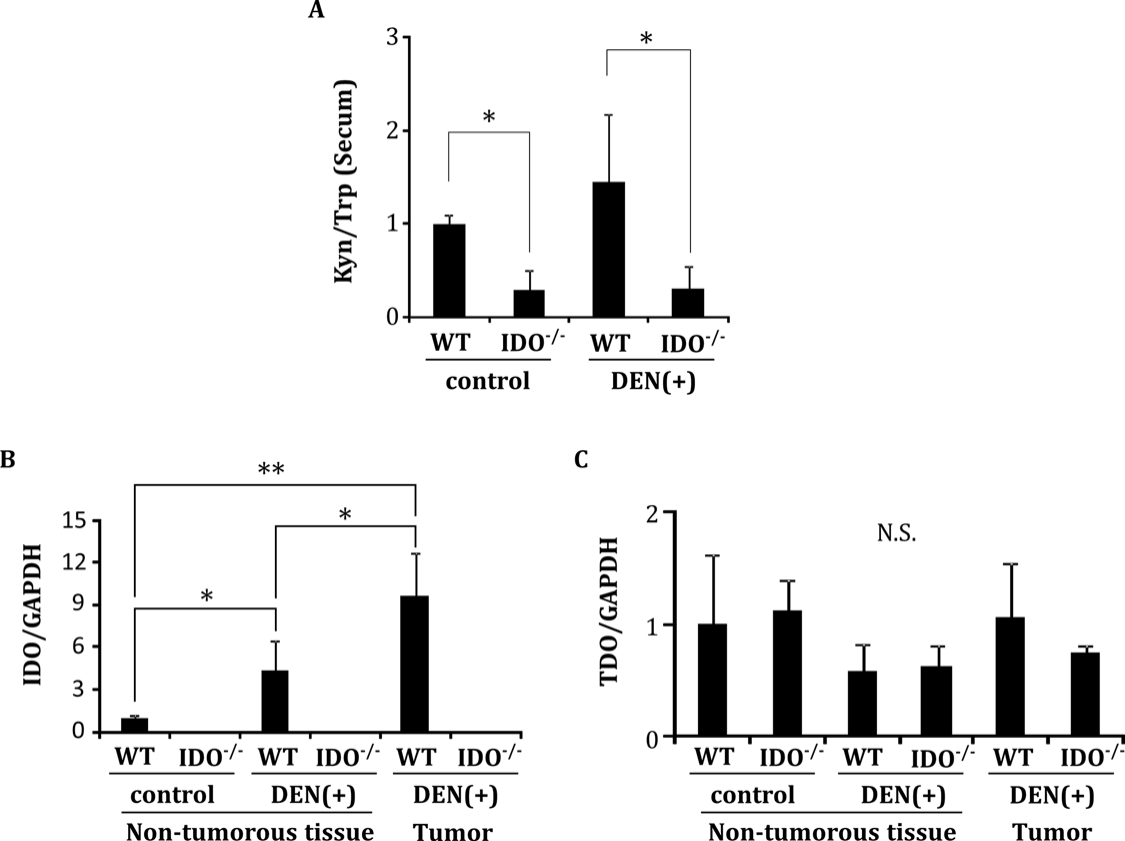
The serum L-kynurenine/L-tryptophan ratio and tissue gene expression of IDO and TDO. **A,** The serum L-kynurenine/L-tryptophan ratio (Kyn/Trp) was determined at 32 weeks by measuring the concentrations of L-kynurenine and L-tryptophan using HPLC. (B, C) Liver tissues were separated into hepatic tumors and non-tumorous tissues. Total RNA was then extracted and the expression levels of IDO and TDO mRNA were measured using quantitative RT-PCR. Data points represent the mean ± SD. * p<0.05, ** p<0.01, N.S.: not significant

There was no significant difference between the DEN-treated WT-group and the WT-control group in the serum L-kynurenine/L-tryptophan ratio, which reflects the enzymatic activity of IDO, at 32 weeks (Fig 6A). However, among IDO-WT mice, the expression levels of IDO mRNA in the hepatic tumors (*P* < 0.001) and surrounding non-tumorous liver (*P* < 0.05) of the DEN-treated group were significantly increased compared to those of the non-tumorous liver of the saline-treated group (Fig 6B). On the other hand, neither DEN treatment nor IDO genotype altered the expression levels of TDO mRNA, a hepatic enzyme that catalyzes the first step of tryptophan degradation, in either liver tumors or non-tumorous liver (Fig 6C).

## Discussion

In the present study, the development of FCA, which are preneoplastic liver cell lesions, and HCCs was significantly increased in the IDO-WT mice as compared to the IDO-KO mice when they received DEN. Liver cell adenomas in the IDO-WT mice showed a large increase in the expression levels of IDO and L-kynurenine, which reflects the enzymatic activity of IDO, compared to the surrounding non-tumorous tissue. Furthermore, the expression levels of IDO and L-kynurenine were also elevated in HCC compared to non-cancerous tissues. These results, therefore, provide the first evidence that expression and activation of IDO is possibly associated with both the early and late phases of liver carcinogenesis.

What key mechanisms accelerate liver tumorigenesis in IDO-WT mice? We presume that increased expression of L-kynurenine, a toxic tryptophan catabolite produced by IDO, is critically involved in this acceleration because this amino acid plays a role in the immune escape of malignant cells that occurs within the tumor and also in the surrounding microenvironment [10, 38]. Elevation of L-kynurenine levels by IDO overexpression facilitates tumor-induced immune tolerance by suppressing cytotoxic T lymphocyte proliferation [39]. Therefore, in the IDO-WT mice, increased expression of IDO and L-kynurenine in liver cell adenomas compared to the surrounding normal tissue may create a microenvironment that promotes the progression of HCC, at least in part by suppressing the proliferation of cytotoxic T lymphocytes. Indeed, the expression levels of CD8, perforin, and granzyme B mRNA were markedly decreased in hepatic tumors in IDO-WT mice as compared to IDO-KO mice (Fig 5, *P* < 0.05).

In addition to direct suppression of cytotoxic T lymphocytes, IDO contributes to tumor-induced immune tolerance by enhancing Tregs, which are major components of the immune suppressive tumor microenvironment [6, 39, 40]. In the present study, Foxp3-positive inflammatory cells, which are regarded as Tregs [37], significantly infiltrated and formed foci in the liver of DEN-treated IDO-WT mice, whereas these cells did not form foci in the livers of DEN-treated IDO-KO mice. The expression levels of Foxp3 mRNA were also notably elevated in the DEN-induced hepatic tumors of IDO-WT mice compared to IDO-KO mice. In addition, clinical studies have shown an increase in IDO in HCC tissues [24] and an elevation of the Treg population in both the periphery and tumor-infiltrating lymphocytes in patients with HCC [41, 42]. These reports, together with the results of the present study, suggest that suppression of tumor immunity by IDO-induced Tregs might play a role in the development and progression of HCC.

Recent clinical and animal studies have demonstrated that IDO expression is significantly enhanced during hepatic inflammation [25, 43]. IDO plays a critical role in the induction of immune escape and inflammation in the inflammatory tissue microenvironment [44, 45]. These reports seem to be significant because such a microenvironment is strongly associated with the development and progression of HCC [46]. IDO is induced during the inflammatory process by several immune factors, including IFN-γ and TNF-α [47, 48]. IFN-γ may function as a tumor promoter by stimulating the release of inflammatory mediators such as TNF-α that advance carcinogenesis [49]. Stimulation with IFN-γ induces IDO gene expression in human HCC-derived cells [24]. COX-2 is also regarded as one of the most critical inflammatory mediators in the regulation of IDO expression [50]. Therefore, in the present study, overexpression of IFN-γ, TNF-α, and COX-2 mRNA might accelerate IDO induction and L-kynurenine elevation in hepatic tumors, which subsequently enhances tumor-induced immune tolerance in the livers of IDO-WT mice. The importance of IDO and COX-2 expression in the development of cancer-associated inflammation has been previously reported [45, 51]. A recent study also demonstrated that IDO and prostaglandin E_2_, one of the products of COX-2, derived from HCC-activated fibroblasts suppress NK cell activation and cytotoxicity, leading to the creation of a favorable microenvironment for tumor development and growth [51]. In the present study, RT-PCR experiments showed that the mRNA expression of proinflammatory cytokines and COX2 was decreased in IDO-KO mice relative to the IDO-WT mice (Fig 3A, 3B and 3C). However, there was no significant difference in their expression in non-tumorous tissue (Fig 3A, 3B and 3C). We previously reported a similar phenomenon in hepatitis B virus (HBV) transgenic/IDO-KO mice in a HBV-specific cytotoxic T lymphocyte induced fulminant hepatitis model [52]. The difference in infiltrating cell number between IDO-WT and IDO-KO mice might contribute to the difference in inflammatory cytokine expression in tumor tissue. The reduction in the expression of these cytokines and COX2 in the tumor tissue indicated that inflammation was decreased in the hepatic tumor tissue of IDO-KO mice. In general, increased hepatic IDO expression induces apoptosis in activated T cells and NK cells [10]. In IDO-WT mice, IDO expression might lead to cell and/or tissue injuries by metabolite such as L-kynurenine, and these injuries might enhance tissue inflammation.

The results of the present study demonstrated that the IDO-KO mice have a reduced susceptibility to DEN-induced liver carcinogenesis. These findings strongly suggest the possibility that targeting IDO and reducing the amount of L-kynurenine might be effective strategies for the treatment and possible chemoprevention of HCC. Several preclinical studies have demonstrated the anti-tumor effects of IDO inhibitor-based combination therapy [20, 21]. Reduction of the kynurenine levels in the microenvironment by using IDO inhibitors impedes the growth of IDO-expressing tumors [53]. In addition, up-regulation of IDO is possibly involved in lymphoma or colon carcinogenesis, whereas treatment with 1-MT, an IDO inhibitor, effectively suppresses chemically induced lymphoma or colorectal carcinogenesis by inhibiting IDO activity [22, 23]. (**−**)-Epigallocatechin gallate, one of the green tea catechins that can decrease IFN-γ-induced IDO expression in colon cancer cells [54], also suppresses colorectal carcinogenesis through the inhibition of IDO activity and COX-2 expression [22]. To test the potential efficacy of IDO inhibitors in the prevention of HCC, additional experiments to evaluate whether these agents, including 1-MT, can suppress liver tumorigenesis associated with IDO overexpression should be conducted.

In summary, we demonstrated in the current study that IDO up-regulation may contribute to the development and progression of liver carcinogenesis, which might be associated with the induction of both inflammation and an immunosuppressive microenvironment. Our findings, together with the results of clinical reports [24, 25, 43], strongly suggest that inhibition of IDO activity might lead to the development of new treatments for chronic liver disease as well as strategies to prevent HCC.
